# Sudden unexpected death in epilepsy: An Italian expert consensus statement on risk, mechanisms, prevention, and therapeutic strategies

**DOI:** 10.1002/epi4.70358

**Published:** 2026-10-07

**Authors:** Vincenzo Belcastro, Fedele Dono, Giovanni Falcicchio, Lorenzo Gigli, Angela La Neve, Sara Matricardi, Barbara Mostacci, Angelo Pascarella, Leandro Pecchia, Emilio Russo, Pasquale Striano

**Affiliations:** ^1^ Neurology Unit Ospedale Maggiore, ASST Lodi Lodi Italy; ^2^ Department of Neuroscience, Imaging and Clinical Sciences “G. d'Annunzio” University of Chieti‐Pescara Chieti Italy; ^3^ Department of Translational Biomedicine and Neuroscience University of Bari “Aldo Moro” Bari Italy; ^4^ Division of Electrophysiology, Cardiology 3 Ospedale Maggiore Niguarda Milan Italy; ^5^ DiBraiN Department University of Bari “Aldo Moro” Bari Italy; ^6^ Department of Pediatrics “G. d'Annunzio” University of Chieti‐Pescara Chieti Italy; ^7^ IRCCS Istituto delle Scienze Neurologiche di Bologna Bologna Italy; ^8^ Department of Medical and Surgical Sciences Magna Græcia University Catanzaro Italy; ^9^ Regional Epilepsy Center Grande Ospedale Metropolitano “Bianchi‐Melacrino‐Morelli” Reggio Calabria Italy; ^10^ Biomedical Engineering Università Campus Bio‐Medico di Roma Rome Italy; ^11^ CRUISE Research Center Department of Health Sciences University Magna Græcia of Catanzaro Catanzaro Italy; ^12^ IRCCS Istituto Giannina Gaslini Genova Italy; ^13^ Department of Neurosciences, Rehabilitation, Ophthalmology, Genetics, Maternal and Child Health University of Genova Genova Italy

**Keywords:** developmental and epileptic encephalopathies, pathophysiology, prevention, risk factors, SUDEP

## Abstract

**Objective:**

Sudden unexpected death in epilepsy (SUDEP) is a leading cause of premature mortality in people with epilepsy, particularly in those with tonic–clonic seizures (TCSs), developmental and epileptic encephalopathies (DEEs), and drug‐resistant epilepsy. Although awareness of SUDEP has increased, major uncertainties remain regarding individualized risk stratification, mechanistic interpretation, and implementation of preventive strategies in routine care. This Italian expert consensus statement was developed to formalize a shared multidisciplinary position on SUDEP risk profiles, pathophysiological mechanisms, and preventive and therapeutic strategies.

**Methods:**

The consensus was supported by a focused narrative appraisal of current evidence, rather than a systematic review, and developed through a structured multidisciplinary process based on a nominal group technique. The panel comprised 11 Italian experts: nine epileptologists from the Italian League Against Epilepsy (LICE) and its SUDEP Working Group, one cardiologist with expertise in epilepsy‐related cardiac complications and SUDEP, and one biomedical engineer with expertise in seizure‐detection devices and wearable technologies. Statements were generated across three macro‐areas, discussed in working groups and plenary sessions, prioritized through an initial in‐person vote, refined after discussion, and submitted to a second online round. Consensus was predefined as at least 75% agreement on a 5‐point Likert scale.

**Results:**

A final set of 33 statements was generated, of which 26 reached consensus; two of these (serotonergic modulation as a preventive strategy and fenfluramine's mechanisms beyond seizure control) reached the threshold only narrowly and are discussed as hypothesis‐generating rather than as established clinical guidance. The resulting framework supports an extension of seizure‐centered prevention toward an integrated, individualized, and mechanism‐informed approach, in which reduction of convulsive seizure burden remains central while post‐ictal respiratory, autonomic, cardiac, arousal, genetic, and treatment‐related vulnerabilities are considered in selected high‐risk patients.

**Significance:**

This consensus provides the first formalized Italian multidisciplinary position on SUDEP, offering an evidence‐informed and mechanism‐aware framework intended to support more consistent risk communication, individualized risk stratification, and preventive care in clinical practice.

**Plain Language Summary:**

Sudden unexpected death in epilepsy (SUDEP) is uncommon but is more likely in people with frequent tonic–clonic seizures and some severe epilepsy syndromes. An Italian multidisciplinary expert panel developed consensus statements on SUDEP risk, possible mechanisms, monitoring, and prevention. Reducing convulsive seizures and supporting treatment adherence remain the main preventive priorities; SUDEP information should be offered broadly, while counseling and additional measures should be tailored to individual risk. These statements reflect expert agreement and available evidence and are not formal clinical practice guidelines.

**Key points**

- Frequent tonic–clonic seizures remain the strongest modifiable marker of SUDEP risk.
- Reducing convulsive seizure burden and supporting adherence are central to SUDEP prevention.
- Post‐ictal respiratory, autonomic, cardiac, and arousal dysfunction may converge.
- SUDEP information should be offered broadly, tailoring counseling depth, timing, and repetition to individual risk.
- Monitoring and cardiological assessment should be individualized; evidence for direct SUDEP prevention remains limited.

## 1 INTRODUCTION

Sudden unexpected death in epilepsy (SUDEP) is defined as the sudden, unexpected, witnessed or unwitnessed, non‐traumatic and non‐drowning death of a person with epilepsy, with or without evidence of a seizure, excluding documented status epilepticus, in whom post‐mortem examination does not reveal a toxicological or anatomical cause of death.^1^, ^2^ Although relatively infrequent at the individual level, SUDEP represents one of the most devastating epilepsy‐related outcomes and a major contributor to premature mortality in people with epilepsy.^1^, ^2^, ^3^, ^4^

The burden of SUDEP is not evenly distributed across the epilepsy population. The most robust clinical predictor is the presence and frequency of tonic–clonic seizures (TCSs), particularly when seizures are nocturnal or unwitnessed.^1^, ^2^, ^5^ Epidemiological data also indicate increased vulnerability in people with drug‐resistant epilepsy and in severe DEEs, particularly Dravet syndrome (DS) and, within a broader high‐risk clinical framework, Lennox–Gastaut syndrome (LGS). In these patients, convulsive seizure burden may coexist with syndrome‐specific biological vulnerability and complex comorbidity.^3^, ^5^, ^6^, ^7^, ^8^

For this consensus, “high‐risk patients” is described using a tiered structure to avoid indiscriminately including most drug‐resistant patients. The highest risk could be, in particular, defined as: frequent or recent nocturnal/unwitnessed TCSs, DEEs (DS, LGS). In addition, risk‐modifying factors include: drug resistance, intellectual disability, poor adherence, limited supervision, cardiorespiratory/genetic markers.^1^, ^2^, ^5^, ^6^

The pathophysiology of SUDEP remains only partially understood. Current evidence supports a multifactorial model in which seizure‐related dysfunction of respiratory, autonomic, cardiac, and arousal networks leads to progressive post‐ictal cardiorespiratory collapse.^2^, ^9^, ^10^ Available monitored cases, particularly the MORTEMUS study, suggest a typical sequence after a TCS, frequently during sleep, followed by post‐ictal respiratory dysfunction, autonomic instability, bradyarrhythmia, and terminal asystole.^9^, ^11^

Accordingly, SUDEP can be conceptualized as a network‐based post‐ictal failure: a seizure‐triggered breakdown of interacting brainstem, respiratory, autonomic, cardiac, and arousal systems rather than the consequence of a single isolated respiratory or cardiac mechanism.^9^, ^10^, ^12^, ^13^

Within this framework, the post‐ictal window represents a crucial time for prevention after a potentially dangerous seizure, because supervision, repositioning, stimulation, rescue medication when clinically indicated, and targeted cardiorespiratory monitoring may act on a still evolving and potentially reversible collapse cascade.^5^, ^12^, ^13^, ^14^ This framing does not displace seizure reduction, surgical or neuromodulatory options, dietary therapy when appropriate, and optimized antiseizure treatment as primary preventive targets; rather, it identifies the post‐ictal phase as an actionable interval once a high‐risk seizure has occurred.

Further progress in SUDEP prevention will require prospective validation of preventive strategies and better translation of mechanistic insights into therapeutic decision‐making.^2^, ^5^, ^15^ Despite existing international guidance, implementation of SUDEP communication, individualized risk assessment, monitoring, and preventive strategies remains heterogeneous in routine care, and a structured Italian multidisciplinary position integrating epilepsy, cardiology, and monitoring perspectives has been lacking. Against this background, this expert consensus statement aims to formalize a shared Italian position on SUDEP clinical features, mechanisms, and preventive and therapeutic strategies, complementing rather than replacing existing international recommendations.

## 2 METHODS

The SURE (Statement & Unified Recommendations by Experts) Working Group was convened as a multidisciplinary Italian expert panel to develop consensus on key clinical and research aspects of SUDEP. The panel comprised 11 experts: nine epileptologists affiliated with LICE and/or its SUDEP Working Group, one cardiologist with specific expertise in epilepsy‐related cardiac complications and SUDEP, and one biomedical engineer with expertise in seizure‐detection devices and wearable technologies. Panelists were selected purposively to combine clinical, scientific, and technological expertise relevant to SUDEP, DEEs, cardiology, seizure‐detection devices, and wearable technologies. The initiative was designed to integrate available scientific evidence with expert opinion and to formulate consensus statements across three predefined macro‐areas: clinical characteristics and risk factors, pathophysiological mechanisms, and preventive and therapeutic strategies.

The consensus process, described in Figure 1, was based on a structured nominal group technique (NGT).^16^ Before the meeting, panel members independently reviewed the relevant literature and received a structured list of key questions covering SUDEP epidemiology, risk assessment, pathophysiology, preventive strategies, and future research priorities. These questions served as the basis for subsequent discussions and consensus voting. During the meeting, experts were assigned to working groups corresponding to the three macro‐areas. Within each group, participants first generated preliminary statements considered relevant for clinical practice and future research (silent generation of ideas). These preliminary statements were then presented in plenary session following a round‐robin format and collectively discussed during a clarification phase to refine wording, improve conceptual consistency, and identify overlap or redundancy across statements. Following the plenary discussion, a first in‐person voting round was conducted to establish the priority of the preliminary statements. The statements generated by the working groups were subjected to a structured prioritization process using a ranking approach, in line with the NGT. Each expert was asked, independently and anonymously, to classify each statement into one of three categories: not a priority, important but not a priority, or high priority to be submitted for consensus voting. This step was aimed at selecting the statements considered most relevant by the panel for inclusion in the subsequent consensus phase, which consisted of agreement rating using a 5‐point Likert scale.

**FIGURE 1:**
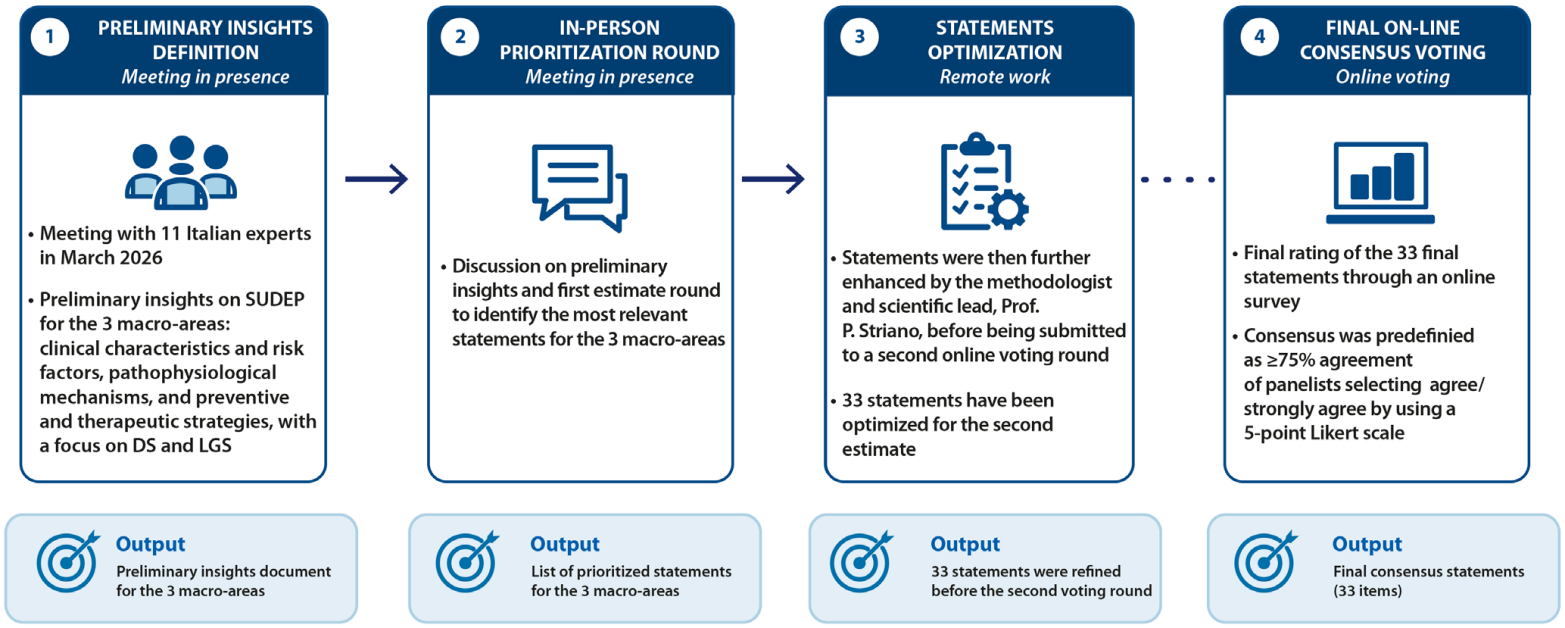
Consensus process based on a structured nominal group technique (NGT), including an initial in‐person prioritization round followed by statement refinement and a final online consensus voting round. DS, Dravet syndrome; LGS, Lennox–Gastaut syndrome; SUDEP, sudden unexpected death in epilepsy.

The supporting evidence appraisal was narrative and intended to contextualize the predefined consensus questions rather than to generate formal evidence‐based recommendations. Panel members independently reviewed literature considered relevant to these questions; this step did not use a predefined systematic or database‐specific search protocol. It did not follow a PRISMA process or include duplicate study selection, formal risk‐of‐bias assessment, or evidence grading.

The highest‐priority statements underwent iterative revision to improve clarity, eliminate overlap, and better reflect the available evidence and expert discussions. Following review by the methodologist and the scientific lead, Prof. Pasquale Striano (P.S.), the final wording of each statement was submitted for a second online voting round.

During the in‐person meeting, the panel collectively agreed on the use of a 5‐point Likert scale (strongly disagree, disagree, neither agree nor disagree, agree, strongly agree) to assess the level of agreement, as it is widely used in consensus processes and considered appropriate to capture both disagreement and intermediate positions without introducing excessive complexity. The 5‐point Likert scale was used for the final consensus agreement‐rating phase. Consensus was predefined as at least 75% of respondents rating a statement as either 4 (“agree”) or 5 (“strongly agree”), calculated based on the frequency of these responses over the total number of voters for each statement. Considering that commonly adopted cut‐offs in structured consensus methods range between 66.6% and 80%, the 75% threshold was deemed appropriate given the specialized nature of the topic, the multidisciplinary composition of the panel, and the relatively small number of experts involved. Consensus percentages quantify agreement among participating experts only; they do not indicate certainty of evidence, effect size, or strength of a clinical recommendation.

Given the multidisciplinary composition of the panel, participants were allowed to refrain from voting on statements addressing highly specialized clinical, genetic, or technological aspects outside their primary area of expertise. Consequently, the denominator for agreement varied across items, with 9 to 11 respondents per statement.

Statements addressing invasive cardiac monitoring, genetic channelopathy testing, and device implantation (B4, B7, B8) were generated and voted on with input from a single cardiologist with epilepsy‐specific expertise; broader cardiogenetic or electrophysiological input was not available, and the voting results for these items should therefore be interpreted in light of this limitation.

A formal GRADE evidence‐to‐decision framework was not used as this project was designed as an NGT‐based expert consensus exercise rather than guideline development. Future updates may benefit from a systematic evidence review and a formal evidence‐to‐decision framework.

## 3 RESULTS

The final set included 33 statements across the three predefined macro‐areas; 26 of 33 statements reached the predefined consensus threshold of at least 75% agreement, whereas seven did not reach consensus. The final statements and voting outcomes are reported in Table 1.

**TABLE 1:** Final statements across the three macro‐areas and voting outcomes.

ID	Statements	Respondents (*n*)	Consensus (%)
A1	DEEs with a high burden of convulsive seizures, particularly Dravet syndrome and Lennox–Gastaut syndrome, represent a high‐risk SUDEP population and require dedicated, proactive preventive strategies	10	100.0%
A2	The main unmet needs in SUDEP prevention are the lack of validated predictive biomarkers and integrated clinical‐genetic risk stratification models	10	100.0%
A3	In SUDEP prevention, risk factors not strictly related to seizures are often underestimated	10	100.0%
A4	SUDEP remains insufficiently recognized outside epilepsy‐specialist settings	11	100.0%
A5	Poor SUDEP awareness may facilitate behaviors associated with higher modifiable risk in people with epilepsy	11	*72.7%*
A6	Relatives and caregivers of people at high SUDEP risk should be trained in post‐ictal supervision, stimulation, and basic resuscitation measures, including in the home setting	10	100.0%
A7	The use of a pulse oximeter and monitoring of vital parameters must always be recommended to relatives of people with epilepsy in the post‐ictal phase	11	*63.6%*
A8	The use of wearable or monitoring devices may create a false perception of protection from SUDEP risk if not integrated with active supervision and emergency response	11	*63.6%*
B1	The immediate post‐ictal period represents a life‐saving window, and targeted post‐ictal cardiorespiratory monitoring should be prioritized in high‐risk patients with convulsive seizures	11	100.0%
B2	Post‐ictal heart‐rate monitoring should be considered in patients with convulsive seizures, with priority in those at higher SUDEP risk	10	80.0%
B3	Clinical signs suggesting a fragile cardiorespiratory phenotype in DS/LGS may include interictal or post‐ictal ECG abnormalities and altered heart‐rate variability	9	88.9%
B4	Genetic testing should be integrated into SUDEP risk assessment in patients with drug‐resistant epilepsy, especially DEEs, and may be expanded to include cardiac channelopathy genes when clinical or electrocardiographic abnormalities suggest shared neurocardiac vulnerability	10	100.0%
B5	A structured cardiological evaluation, including interictal 12‐lead ECG, Holter‐ECG, and post‐ictal ECG when feasible, should be considered in selected high‐risk patients	10	100.0%
B6	In patients with abnormalities on ECG, post‐ictal ECG, or Holter monitoring, 12‐lead ECG evaluation during video‐EEG monitoring may be appropriate	9	88.9%
B7	In patients with relevant abnormalities on ECG, post‐ictal ECG, or Holter monitoring, implantation of a loop recorder may be considered after multidisciplinary evaluation	9	*66.7%*
B8	Invasive cardiac monitoring is mandatory in selected patients with genetically defined increased risk after multidisciplinary assessment	9	*44.4%*
B9	Serotonergic modulation represents a biologically plausible strategy for SUDEP prevention	9	77.8%
Ca1	From a SUDEP prevention perspective, treatment optimization should aim not only at reducing seizure frequency but at achieving the maximal possible reduction in convulsive seizure burden, severity, and duration, including rational polytherapy based on complementary mechanisms of action in high‐risk patients	9	100.0%
Ca2	Within a multimodal approach for SUDEP, beta‐blockers may be considered in selected cases following an individualized multidisciplinary evaluation	10	*30.0%*
Ca3	Fenfluramine may contribute to SUDEP risk reduction through mechanisms that extend beyond seizure control and may involve a direct action on brainstem cardiorespiratory pathways	9	77.8%
Ca4	Fenfluramine represents a valuable therapeutic option for patients with DEEs when indicated, particularly in high‐risk forms where its clinical efficacy and mechanistic profile are especially relevant	9	88.9%
Cb1	When poor or unstable adherence is suspected, proactive communication of SUDEP risk should be used as part of a multidisciplinary strategy to improve treatment adherence and to avoid inappropriate therapeutic de‐escalation in high‐risk patients	9	100.0%
Cb2	In patients at increased SUDEP risk, including those with DEEs such as Dravet syndrome and Lennox–Gastaut syndrome, SUDEP risk should be routinely and explicitly discussed, independently of treatment strategy or adherence status, through proactive, individualized, and family‐centered counseling starting at diagnosis and revisited over time	9	88.9%
Cb3	The integrated use of AI‐supported wearables may be considered in selected high‐risk patients, but is not universally indicated	11	90.9%
Cb4	Night‐time supervision should always be considered in high‐risk patients, particularly those with nocturnal or convulsive seizures	10	100.0%
Cb5	The minimal set of nocturnal monitoring systems should include a real‐time caregiver alert; oxygen saturation, heart rate, and movement‐based signals may be integrated according to clinical context and device feasibility	10	90.0%
Cb6	The use of an anti‐suffocation pillow is advised in all patients with convulsive/nocturnal seizures	10	*70.0%*
Cb7	The minimum post‐ictal management package should include prompt administration of rescue therapy when clinically indicated	10	90.0%
Cb8	The adoption of AI tools in seizure monitoring should aim for an optimal balance between sensitivity, specificity, and clinical usability	10	90.0%
Cb9	The minimum standard for adoption of wearables in clinical practice should include reliable event recording, minimal patient discomfort, and reduced caregiver anxiety	10	90.0%
Cb10	Wearable devices should be considered primarily in patients with active epilepsy, with priority for those at higher SUDEP risk	10	90.0%
Cb11	When monitoring devices are used, preference should be given to multimodal systems capable of detecting a broad range of ictal phenomena and that are economically sustainable in routine care	10	100.0%
Cb12	The development of prospective registries will allow better identification of clinically meaningful high‐risk subpopulations	10	100.0%

*Note*: Consensus was predefined as at least 75% of respondents rating a statement as either agree or strongly agree on a 5‐point Likert scale. The number of respondents varied across statements because panelists were allowed to abstain from voting on highly specialized items outside their primary area of expertise; therefore, the denominator for agreement was specific to each item. Consensus percentages quantify agreement among participating experts only and do not indicate certainty of evidence, effect size, or strength of a clinical recommendation. These statements are expert consensus outputs and should not be interpreted as guideline‐level recommendations. Statements reaching less than 75% consensus are highlighted in the table. Italic values indicate statements that did not reach the predefined 75% consensus threshold.

Abbreviations: AI, artificial intelligence; DEE, developmental and epileptic encephalopathy; DS, Dravet syndrome; ECG, electrocardiogram; LGS, Lennox–Gastaut syndrome.

The voting pattern also identified the statements that generated greater debate and, therefore, require cautious interpretation. Consensus was not reached for poor SUDEP awareness as a direct driver of modifiable risk behaviors (A5), universal post‐ictal pulse oximetry or vital‐parameter monitoring for relatives (A7), the assumption that wearables necessarily create a false sense of protection (A8), loop recorder implantation after ECG or Holter abnormalities (B7), mandatory invasive cardiac monitoring in genetically high‐risk patients (B8), beta‐blockers as part of a multimodal SUDEP‐prevention strategy (Ca2), and universal use of anti‐suffocation pillows in patients with convulsive or nocturnal seizures (Cb6). Statements on serotonergic modulation (B9) and fenfluramine mechanisms beyond seizure control (Ca3) reached consensus only narrowly and are therefore discussed as biologically plausible and hypothesis‐generating rather than as direct clinical evidence of SUDEP prevention.

## 4 DISCUSSION

### 4.1 SUDEP clinical profile and high‐risk populations

The first macro‐area reframes SUDEP as a major epilepsy‐related outcome with discernible patterns of risk across identifiable clinical subgroups, rather than as a rare and largely unpredictable complication. DEEs with a high burden of convulsive seizures, particularly Dravet syndrome and, within a broader high‐risk clinical framework, Lennox–Gastaut syndrome, emerge as priority settings for proactive prevention. The evidence base is stronger for Dravet syndrome, whereas SUDEP‐specific data in Lennox–Gastaut syndrome remain more limited despite its high mortality and clinical complexity.^1^, ^2^, ^3^, ^6^ In addition, a recent Bayesian meta‐analysis of 37 RCTs and 15 extension studies (3757 patients) estimated an overall SUDEP incidence of 4.32/1000 person‐years in DEEs, with the highest rate observed in Dravet syndrome (7.59/1000 person‐years) and a substantial risk also in Lennox–Gastaut syndrome (3.40/1000 person‐years), confirming that SUDEP represents approximately half of all premature deaths in these disorders.^17^

The panel also converged on two major unmet needs: the absence of validated predictive biomarkers and the lack of integrated clinical‐genetic models for individualized risk stratification. Current risk assessment still depends largely on clinical history, seizure burden, nocturnal occurrence, adherence, supervision, and comorbidity, while genetic information is increasingly relevant but not yet prospectively validated as a stand‐alone SUDEP predictor.^1^, ^2^, ^18^

The voting results reinforce seizure control as the principal modifiable preventive target while highlighting nocturnal risk, supervision, treatment adherence, syndrome‐specific vulnerability, and post‐ictal recovery as clinically relevant contextual modifiers (Box 1).^1^, ^2^, ^3^, ^5^, ^6^, ^19^ These domains should be interpreted together rather than as independently validated predictors.

BOX 1 **Multidimensional SUDEP prevention model**

**Table:** 

Core target: reduce TCS burden through optimized antiseizure treatment and adherence. Contextual risk assessment: nocturnal seizures, supervision, intellectual disability, prone post‐ictal position,^19^ and syndrome‐specific vulnerability. Post‐ictal response: caregiver alert, repositioning or stimulation, rescue medication when clinically indicated, and targeted monitoring in selected high‐risk patients. Research priorities: validated biomarkers, integrated clinical‐genetic risk models, and prospective registries.^1^, ^2^, ^5^, ^6^, ^14^, ^15^

Published literature supports SUDEP counseling as part of good clinical care,^1^, ^20^, ^21^ whereas the panel vote specifically addressed implementation in higher‐risk settings. The lack of consensus that poor awareness alone directly promotes higher‐risk behaviors should therefore not be interpreted as evidence against informing lower‐risk or newly diagnosed patients. Counseling should remain individualized, family‐centered, and embedded in broader educational, therapeutic, and support pathways rather than treated as a stand‐alone behavioral intervention.

A similar caution applies to monitoring and device‐related statements. Lack of consensus on universal post‐ictal pulse oximetry or vital‐parameter monitoring should not be read as evidence against selected use. Rather, in line with the available literature, the panel favored risk‐adapted monitoring in high‐risk contexts and avoided universal recommendations or deterministic assumptions about device‐related behavior.^5^, ^14^, ^15^ Taken together, the first macro‐area combines a clear focus on high‐risk populations with appropriate caution where direct outcome evidence is limited.

### 4.2 Pathophysiology and involved mechanisms

The statements in the second macro‐area are consistent with the current interpretation of SUDEP as the result of dynamic interaction among seizure activity, respiratory control, cardiovascular regulation, autonomic function, and impaired post‐ictal recovery. Rather than treating respiratory, autonomic, and cardiac abnormalities as isolated phenomena, the panel interpreted them as interconnected components of a temporally linked post‐ictal cascade, in line with the sequence of events described in monitored SUDEP cases.^2^, ^9^, ^10^, ^11^, ^12^, ^13^, ^14^

Within this framework, the immediate post‐ictal period emerges as a critical window of vulnerability. The panel reached consensus on the importance of targeted post‐ictal cardiorespiratory monitoring in high‐risk individuals with convulsive seizures and considered post‐ictal heart‐rate monitoring a reasonable component of a broader surveillance strategy. These statements are clinically important because they emphasize the post‐ictal period rather than the seizure alone, as evidence from monitored SUDEP cases suggests that this is the phase in which the fatal sequence most clearly unfolds. The implication is not that monitoring alone prevents SUDEP, but that the post‐ictal window is the phase in which deterioration may be recognized and response pathways can still be activated.^5^, ^12^, ^13^, ^14^

Respiratory dysfunction remains central to the model, while cardiac abnormalities may act as downstream effectors, co‐determinants, or markers of broader cardiorespiratory vulnerability in susceptible individuals. Overall, the evidence supports an integrated model involving brainstem, autonomic, respiratory, and cardiac networks rather than a single isolated mechanism.^10^, ^12^, ^13^, ^22^

The importance given to the cardiological dimension is reflected in the statements supporting structured cardiological evaluation in selected high‐risk patients, including interictal 12‐lead ECG, Holter‐ECG, post‐ictal ECG when feasible, and ECG evaluation during video‐EEG monitoring when prior abnormalities are present. These findings reflect expert opinion rather than trial‐derived evidence and support consideration of a risk‐stratified approach to cardiological evaluation in selected high‐risk patients, pending prospective validation; non‐invasive assessment is endorsed as a reasonable first step, while more advanced investigations should be considered on an individualized multidisciplinary basis when clinical context, electrocardiographic abnormalities, or genetic findings raise concern.^2^, ^12^, ^23^, ^24^, ^25^

The statement on genetic testing recognizes that, particularly in drug‐resistant epilepsies and DEEs, etiological genetic diagnosis may provide clinically relevant information beyond syndrome classification. In selected patients, it may help contextualize potential neurocardiac vulnerability, an area supported by emerging evidence on genes expressed in both the central nervous system and the cardiovascular system. However, the current role of genetic findings in prospective SUDEP risk prediction remains incompletely defined.^2^, ^13^, ^25^

Finally, the panel reached only narrow consensus on serotonergic modulation (B9). Published preclinical and mechanistic literature provides biological plausibility for an influence on post‐ictal respiratory recovery and seizure‐induced respiratory arrest,^9^, ^10^, ^12^, ^26^ but direct clinical confirmation is limited. This statement should therefore be regarded as hypothesis‐generating and is not sufficient to support clinical guidance for SUDEP prevention.

Taken together, the second macro‐area distinguishes mechanistic plausibility from clinical implementation: post‐ictal monitoring and selected cardiological assessment may be considered in higher‐risk phenotypes, while genetic and serotonergic hypotheses require prospective validation before they can inform broader practice.

### 4.3 Therapeutic strategies, preventive care, and future directions

#### 4.3.1 Therapeutic strategies

The strongest area of agreement is that SUDEP risk reduction currently relies mainly on reducing the burden, severity, and duration of TCSs, particularly in high‐risk patients. This approach is consistent with the most robust evidence currently available and should include optimized syndrome‐informed antiseizure treatment and, when appropriate, surgical, neuromodulatory, dietary, and rescue strategies.^1^, ^5^

Within pharmacological strategies specifically discussed for SUDEP prevention, beta‐blockers were evaluated as a potential multimodal option. Consensus was not reached on this statement (Ca2); their use should therefore remain individualized and restricted to specific neurocardiological contexts rather than presented as broadly applicable.^2^, ^5^

Among the therapeutic options considered, fenfluramine (FFA) is discussed as two consensus statements that specifically addressed this agent (Ca3 and Ca4). FFA has an established antiseizure role in clinically appropriate patients with DS and LGS, with randomized and long‐term evidence showing clinically meaningful reductions in seizure burden.^27^, ^28^, ^29^, ^30^, ^31^ In addition, emerging evidence suggests potential benefits on selected non‐seizure outcomes in these syndromes.^32^ Beyond these clinical effects, FFA has a distinctive multimodal mechanism of action involving serotonergic pathways and sigma‐1 receptor modulation.^33^, ^34^ This provides a biologically plausible rationale for a potential influence on SUDEP‐related vulnerability, further supported by preclinical evidence showing that FFA can reduce seizure‐induced respiratory arrest in a mouse model of SUDEP.^35^ In addition, pooled Dravet syndrome exposure data reported a lower all‐cause/SUDEP mortality rate during FFA treatment (1.7 per 1000 person‐years) than historical estimates.^36^ Together, these mechanistic, preclinical, and clinical observations support further investigation of a potential role of FFA in SUDEP‐related pathways. However, the evidence remains hypothesis‐generating, and the potential role of FFA in SUDEP prevention requires prospective confirmation.

#### 4.3.2 Preventive care pathways and monitoring technologies

Published guidance and recent communication evidence support offering SUDEP information broadly to people with epilepsy and their families.^1^, ^21^ The panel identified the need for proactive, explicit, individualized, and repeated counseling in patients at increased risk, as determined by clinical evaluation. For patients at lower current risk, SUDEP information should remain available, with the timing, depth, and repetition of discussion tailored to individual circumstances and clinical judgment. Counseling should remain family‐centered and integrated with adherence support, rescue planning, supervision, and individualized risk assessment.

Selective use of seizure‐detection devices, AI‐supported tools, wearables, and multimodal monitoring may be appropriate in patients with active epilepsy and higher SUDEP risk, particularly those with recurrent convulsive seizures, when the goal is to improve detection, supervision, and timely response.^5^, ^15^, ^23^ Here, active epilepsy refers to ongoing seizures despite treatment or recurrent clinically relevant seizures requiring continuing management. Wearable and AI‐supported technologies should be interpreted primarily as tools to improve detection and response in selected high‐risk patients, rather than as stand‐alone SUDEP‐preventive interventions.

Night‐time supervision should be discussed with patients with nocturnal or convulsive seizures. By contrast, no consensus was reached regarding the routine use of anti‐suffocation pillows. The panel recognized that their potential role in SUDEP risk mitigation still requires stronger supportive evidence and raised concern that such devices could create a false sense of security if used in isolation; therefore, they should not be considered a substitute for established preventive strategies focused on seizure control, supervision, and individualized risk assessment.^5^, ^14^

#### 4.3.3 Future directions

Prospective registries remain a key priority for capturing rare outcomes, defining high‐risk subgroups, and integrating clinical, genetic, physiological, treatment, counseling, and monitoring variables into more refined SUDEP risk models.^2^, ^24^ Because SUDEP is a rare outcome, adequately powered interventional studies using SUDEP as a direct endpoint are difficult to conduct, particularly in rare DEEs. Well‐designed registries and real‐world evidence studies may therefore provide a practical framework for evaluating syndrome‐specific risk modifiers, counseling pathways, monitoring strategies, peri‐ictal interventions, and treatment‐related clinical signals over time.

### 4.4 Clinical relevance

In light of these considerations, the panel's conclusions can be summarized within the multidimensional framework for SUDEP risk and prevention shown in Figure 2. To facilitate clinical interpretation, Box 2 proposes a simple, risk‐informed pathway for SUDEP communication, individualized risk assessment, preventive actions, and periodic reassessment. The pathway is intended as a practical synthesis of main available evidence and panel consensus outputs to support individualized clinical assessment and decision‐making.^1^, ^2^, ^14^, ^21^

**FIGURE 2:**
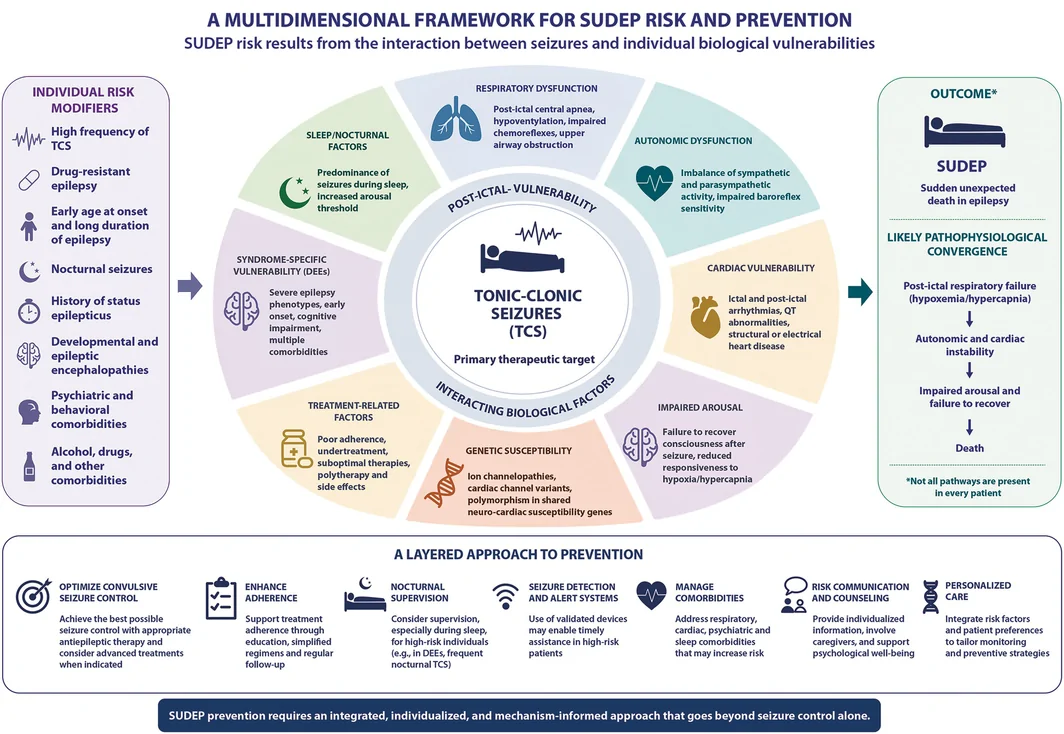
A multidimensional framework for SUDEP risk and prevention. The figure integrates individual risk modifiers, the interacting biological vulnerabilities underlying post‐ictal collapse, and a layered, risk‐adapted approach to prevention. DEE, developmental and epileptic encephalopathy; SUDEP, sudden unexpected death in epilepsy; TCS, tonic–clonic seizure.

BOX 2 **Proposed practical pathway for SUDEP communication and prevention**

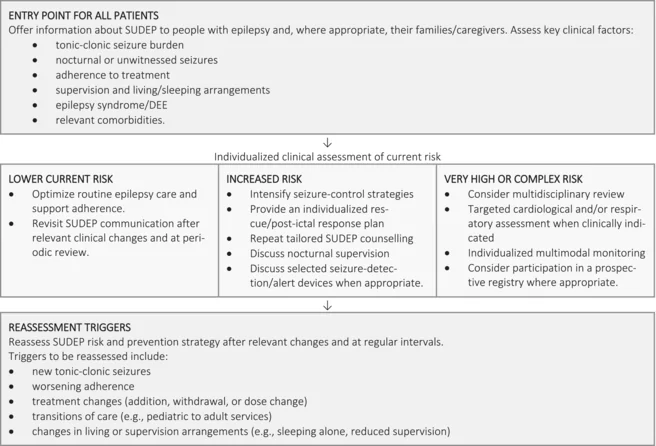

*Information should be offered to all patients and families, while the intensity of counseling, surveillance, and multidisciplinary assessment is tailored to current risk. Monitoring devices do not replace seizure control, adherence, supervision, or an individualized emergency response plan*.

## 5 LIMITATIONS

The present consensus should be interpreted in light of several methodological and contextual considerations. Because SUDEP is a rare outcome, adequately powered prospective and interventional studies using SUDEP as a primary endpoint are challenging to conduct. Many statements therefore draw on observational evidence, biological plausibility, and clinical experience rather than dedicated prospective trials. In addition, the supporting evidence appraisal was narrative rather than systematic, no formal risk‐of‐bias assessment or GRADE evidence grading was performed, and consensus percentages quantify expert agreement rather than certainty of evidence or recommendation strength.

In addition, although the panel included experts from different disciplines and areas of expertise, it was relatively small, entirely Italian, and predominantly epilepsy‐oriented. The predominance of epileptologists and the absence of direct patient and caregiver involvement may limit the generalizability, acceptability, feasibility, and patient‐centeredness of statements concerning counseling, nocturnal supervision, caregiver response, respiratory assessment, and monitoring technologies. These domains would benefit from validation with direct patient and caregiver involvement and broader representation from nursing, psychology, respiratory medicine, genetics/cardiogenetics, health economics, and implementation science.

Furthermore, the statements reflect consensus within a specific national healthcare context and may not fully account for differences in healthcare systems, resources, and clinical practice across countries. Core principles such as seizure control, adherence support, broad provision of SUDEP information, individualized risk assessment, and periodic reassessment are broadly transferable, whereas cardiological pathways, caregiver training, emergency‐response systems, and monitoring technologies should be adapted to local resources and organization of care.

Despite these limitations, the consensus provides a structured framework that integrates current evidence with clinical experience and may support more consistent risk communication, risk stratification, and preventive care. Future prospective registries, multinational collaborative datasets, and real‐world studies will be important to validate the statements, refine risk assessment, and evaluate preventive interventions in routine practice; future consensus updates may also benefit from a systematic evidence review and a formal evidence‐to‐decision framework to more explicitly link evidence certainty, benefits and harms, feasibility, stakeholder values, and recommendation strength.

## 6 CONCLUSIONS

SUDEP remains one of the most serious complications of epilepsy, particularly in individuals with tonic–clonic seizures, drug‐resistant epilepsy, and developmental and epileptic encephalopathies. While seizure control remains the cornerstone of prevention, a broader approach incorporating post‐ictal vulnerability, treatment adherence, individualized counseling, and selected monitoring strategies may further support risk reduction.

This Italian multidisciplinary consensus identifies areas of expert agreement while acknowledging heterogeneity in the supporting evidence. Convulsive seizure control and adherence remain the principal modifiable targets; SUDEP information should be offered broadly, with the intensity of counseling and additional preventive measures tailored to individual risk and clinical context. Cardiological assessment, post‐ictal monitoring, wearable technologies, and mechanism‐based therapeutic hypotheses require selective application and further prospective validation. These consensus statements are not formal clinical practice guidelines and should be interpreted and implemented according to individual clinical circumstances, local resources, and care pathways.

## AUTHOR CONTRIBUTIONS

All authors conceived the project, discussed the results, and contributed equally to the development and finalization of the manuscript. All authors contributed to conceptualization, manuscript discussion during and after the in‐person expert meeting, drafting or critical revision of the manuscript, and approval of the final version.

## FUNDING INFORMATION

No external funding was received for this work. The authors covered the costs of limited methodological and editorial support provided by Content Ed Net srl. Content Ed Net had no role in statement generation, voting, data interpretation, or the decision to submit the manuscript.

## CONFLICT OF INTEREST STATEMENT

Pasquale Striano has received honoraria for advisory boards, consultancy activities, speaker fees, educational activities, travel grants, and research support from pharmaceutical and biotechnology companies active in the fields of epilepsy, neurogenetics, neuromuscular disorders, and rare diseases, including Angelini Pharma, UCB, Jazz Pharmaceuticals, Biogen, PTC Therapeutics, and other companies developing therapies for neurological disorders. He has participated as an investigator, principal investigator, or scientific advisor in industry‐sponsored and academic clinical studies. These relationships are unrelated to the present activity and do not influence his independent scientific and clinical judgment. Emilio Russo has received speaker fees or funding from and has participated on advisory boards for Angelini Pharma, Eisai, Ethypharm, GW Pharmaceuticals, Jazz Pharmaceuticals, Kolfarma, Lundbeck, and UCB Pharma. Giovanni Falcicchio has received speaker fees or funding from Angelini Pharma, Lusofarmaco, Eisai, and UCB Pharma. Vincenzo Belcastro has received speaker fees and has participated on advisory boards for Angelini Pharma, Jazz Pharmaceuticals, Lusofarmaco, UCB Pharma, and Italfarmaco. Angelo Pascarella has received honoraria for consultancy activities from UCB Pharma. Barbara Mostacci has received personal fees from Livanova, Angelini, Eisai, Lusofarmaco, and UCB Pharma. She has served as an investigator in trials for which her institution received compensation from Livanova, Xenon, Takeda, and BioHaven. Angela La Neve has received speaker's or consultancy fees from Eisai, Mylan, Sanofi, Bial, GW, UCB Pharma, Arvelle Therapeutics, Angelini Pharma, Jazz Pharmaceuticals, Lusofarmaco, and Neuraxpharma. Given that several panel members disclose relationships with companies involved in fenfluramine development or commercialization, drug‐specific voting results and consensus margins are reported transparently. Fenfluramine‐specific statements are presented as outputs of the consensus exercise rather than as clinical recommendations for SUDEP prevention, and mechanistic claims remain hypothesis‐generating. No manufacturer participated in statement generation, voting, analysis, interpretation, or manuscript preparation. The other authors report no conflicts of interest. We confirm that we have read the Journal's position on issues involved in ethical publication and affirm that this report is consistent with those guidelines.

## ETHICS APPROVAL STATEMENT

This work is a consensus manuscript without patient‐level data collection, indicating that formal ethics committee approval was not required according to local regulations.

## PATIENT CONSENT STATEMENT

Given the nature of this manuscript, no patients were involved and no patient consent was required.

## Data Availability

For this expert consensus, no new datasets were generated. The reported evidence is based on the literature.
